# Complication Avoidance in Surgical Management of Vertebral Column Tumors

**DOI:** 10.3390/curroncol29030121

**Published:** 2022-02-25

**Authors:** Joshua Feler, Felicia Sun, Ankush Bajaj, Matthew Hagan, Samika Kanekar, Patricia Leigh Zadnik Sullivan, Jared S. Fridley, Ziya L. Gokaslan

**Affiliations:** 1 The Warren Alpert Medical School of Brown University, Providence, RI 02912, USA; joshua.feler1@lifespan.org (J.F.); fsun@lifespan.org (F.S.); ankush_bajaj@brown.edu (A.B.); matthew_hagan@brown.edu (M.H.); samika_kanekar@brown.edu (S.K.); psullivan4@lifespan.org (P.L.Z.S.); jared.fridley@lifespan.org (J.S.F.); 2 Department of Neurosurgery, Rhode Island Hospital, Providence, RI 02903, USA

**Keywords:** patient safety, vertebral column tumors, pseudarthrosis, approach-related morbidity

## Abstract

The surgical management of spinal tumors has grown increasingly complex as treatment algorithms for both primary bone tumors of the spine and metastatic spinal disease have evolved in response to novel surgical techniques, rising complication rates, and additional data concerning adjunct therapies. In this review, we discuss actionable interventions for improved patient safety in the operative care for spinal tumors. Strategies for complication avoidance in the preoperative, intraoperative, and postoperative settings are discussed for approach-related morbidities, intraoperative hemorrhage, wound healing complications, cerebrospinal fluid (CSF) leak, thromboembolism, and failure of instrumentation and fusion. These strategies center on themes such as pre-operative imaging review and medical optimization, surgical dissection informed by meticulous attention to anatomic boundaries, and fastidious wound closure followed by thorough post-operative care.

## 1. Introduction

Extradural spinal tumors are categorized as primary when they arise directly from structures of the spine, and as metastatic when they arrive in the spine or epidural space, usually through hematogenous spread from other organs. The role of surgery in treatment differs between the two: primary spinal tumors are generally resistant to chemotherapy and radiation, and thus surgery for tumors such as chordomas and chondrosarcomas aims at more complete removal through en bloc resection, often requiring collaboration by multiple surgical specialties. Although surgery may be curative for spinal tumors with a single metastasis, operations for spinal metastases are frequently performed with palliative intent. Most metastatic spinal tumors are managed primarily with chemotherapy and radiation, and surgery is reserved for spinal instability to alleviate the mechanical pain. Operative management is termed separation surgery when used to create a safe margin between the spinal cord and the tumor for radiation which might otherwise cause radiation injury to the spinal cord at the doses required to effectively treat the tumor. With the advent of minimally invasive surgical techniques, novel targeted therapies, and newer modes of radiation, the treatment algorithm for spinal tumors is evolving. A detailed understanding of surgical complications must also be considered in designing each patient’s treatment. The tumor’s molecular profile, its location, and its symptomatology impact the goals of surgery, which in turn shape the surgical plan and its risk profile. In our discussion of surgical techniques and complications, we shall describe actionable interventions in the preoperative, intraoperative, and postoperative settings that reduce surgical complications and maximize patient safety.

## 2. Background

Primary extradural spinal tumors are the minority, accounting for 10% or less of all osseous spinal tumors. Chordoma, chondrosarcoma, osteosarcoma and Ewing’s sarcoma are the leading malignant primary spinal tumors, while osteoblastoma, osteoclastoma, giant cell tumor, aneurysmal bone cyst, osteoid osteoma, eosinophilic granuloma, and hemangioma are the most common benign primary spinal tumors [1].

While nearly all types of cancer can metastasize to the spine, the three most common are breast, lung, and prostate cancers. Patients typically present between 40 and 70 years of age with a slight male predominance in a 3:2 ratio [2], thought to be due to the propensity of prostate cancer to metastasize to bone. Lung cancer is overall the most commonly diagnosed spinal metastases, comprising 36% of all spinal metastases. Additional primary tumors with an affinity for bone metastasis include malignant melanoma, renal, gastrointestinal, gynecological, bladder, thyroid, and colorectal tumors [3].

The spine accounts for 70% of all osseous metastases. Within the spine, the most common location for spinal metastasis is the thoracic spine (60–80%), followed by the lumbar spine (15–30%) and then the cervical spine (<10%) [4]. Tumors may be further divided by the layer in the spine: extradural, intradural extramedullary, intradural intramedullary. Extradural tumors are by far the most common, comprising 95% of spinal metastases, while intradural metastases are relatively rare—an estimated 8.5% of all central nervous system metastases, and 2.1% of all cancer patients [4].

Surgical approaches to spine tumor may be generally broken up into en bloc and intralesional resections. The former, commonly used in primary tumors and occasionally in radioresistant metastatic tumors, attempts to remove the entirety of the tumor without violating its capsule. In the latter, commonly used for radio- or chemosensitive tumors or when the surgical morbidity of en bloc resection would be unacceptable, tumor mass is deliberately left behind with plans to further treat the remainder with adjuvant therapies. The superiority of en bloc resection in reducing local recurrence and improving overall survival has been demonstrated in giant cell tumors of spine [5], in chordoma [6], and in chondrosarcoma [7]. For radio-resistant or hormone secreting solitary metastasis such as renal cell carcinoma [8], en bloc resection may also be indicated if it can be achieved with an acceptable amount of surgical morbidity within the context of the patient’s overall prognosis [9].

Unlike in primary tumors, the surgical treatment of multiple spinal metastases is palliative, not curative. The Neurological Oncological Mechanical Systemic (NOMS) criteria are a widely accepted working algorithm for operative decision-making in metastatic spinal tumors [10]. Neurological factors include symptoms from compression of neuronal elements, i.e., radicular pain or myelopathy, and radiographic epidural spinal cord compression (ESCC) as described by the six-point grading system designed and validated by the Spine Oncology Study Group (SOSG).

Oncological considerations include a tumor’s chemosensitivity and radiosensitivity; the latter is defined by tumor response to conventional external beam radiation therapy (cEBRT). Radiosensitive histologies include lymphoma, seminoma, and myeloma; cEBRT treats these tumors effectively in terms of symptomatic relief and satisfactory local control while avoiding damage to and compromise of neural elements. Radiosensitive solid tumor histologies include breast, prostate, ovarian, and neuroendocrine carcinomas. Renal, thyroid, hepatocellular, colon, and non-small cell lung carcinomas, in addition to sarcoma and melanoma, represent radioresistant tumors.

Mechanical instability represents an independent indication for surgical intervention, regardless of spinal cord compression and radiosensitivity, and is described by the 18-point Spinal Instability Neoplastic Score (SINS) [10]. The SINS captures both structural changes due to tumor presence and symptoms which imply mechanical instability; pain worse with weight bearing and axial loading is responsive to surgical restoration of mechanical stability.

Finally, systemic factors include a patient’s overall prognosis, remaining medical treatment options, and ability to tolerate the morbidity associated with surgery. Patients whose prognoses preclude postoperative recovery are not appropriate surgical candidates, and patients with extensive systemic disease burden may have an unfavorable risk profile for medical and surgical complications.

## 3. Discussion

### 3.1. Complication Avoidance

Complications can be categorized into approach-related morbidities, intraoperative hemorrhage, wound healing complications, CSFleak, thromboembolism, and failure of instrumentation and fusion. These risks may be mitigated through careful preoperative planning, meticulous surgical technique, and adherence to standard measures to avoid medical complications. Other perioperative measures that can improve safety include intraoperative navigation and neuromonitoring.

### 3.2. Approach Related Morbidity

Establishing adequate access to resect a tumor—whether en bloc or intralesional—may cause significant trauma to local tissues and may require the sacrifice of nervous or vascular structures. Unintended injury may be avoided by careful review of vascular and magnetic resonance imaging (MRI) preoperatively and understanding the relationship of these structures to the tumor, including any aberrations from typical anatomy.

Intraoperatively, excessive injury to local tissues may be avoided with navigation. Image-guided navigation allows the surgeon to use a pointer to correlate structures in the operative field with previously acquired radiographic images to continuously optimize the operative corridor, avoid vitals structures, and accurately place hardware such as pedicle screws when normal anatomical landmarks might be disrupted.

Neurological morbidity can be minimized with intraoperative neuromonitoring. The consequences of nerve root sacrifice depend on the segment. Disruption of upper cervical roots may cause diaphragmatic paralysis, while damage to lower cervical roots risks significant functional impairment of the upper extremity at the corresponding myotome. Posterior approaches to lumbar spine tumors with ventral involvement should only be performed at or above L2, as sacrifice of L1 and L2 nerve roots permits debulking of the tumor mass without the significant neurologic impairment and loss of ambulation associated with injury to the L3–5 nerve roots [11]. Ligation of the S2 and S3 nerve roots for resection of high sacral neoplasms will often result in urinary and fecal incontinence [11] and is associated with significantly worse patient-reported quality of life metrics compared to lower sacral resections [12]. Nerve root sacrifice in the thoracic spine is of little functional consequence [13]. Intraoperative neuromonitoring allows for testing of individual nerve root functions to reduce post-operative disability by identifying critical nerve roots as some functions may be duplicated or vary between individuals. Unfortunately, these sacrifices may not be avoidable, and preoperative patient counseling about the risks and benefits of these outcomes is imperative.

Each region of the spine presents a different technical challenge. In the cervical spine, vital structures in the anterior compartments include the carotid artery, trachea, esophagus, thyroid gland, and parathyroid glands, and their associated vasculature and nerves. In both anterior and posterior approach, the vertebral arteries and nerve roots are at risk. Unilateral vertebral artery sacrifice can be safely performed if pre-operative angiography of the carotid and vertebral arteries suggests adequate alternative perfusion to the posterior cranial circulation to avoid brainstem or cerebellar infarct [14]. Pre- or intraoperative occlusion testing may be performed to further evaluate the safety of this sacrifice.

The thoracic spine is closely associated with vital structures such as the lungs, aorta, superior and inferior vena cava, mediastinum, and has a tenuous vascular supply. Anterior approaches may require traversing the pleura, requiring chest tube placement after surgery to avoid pneumo- or hemothorax. The primary arterial supply of the thoracic spinal cord, the Artery of Adamkiewicz, has a variable origin though most commonly from segmental arteries in the lower thoracic or upper lumbar level [15]; damage to this artery can infarct the spinal cord and precipitate paraplegia. Collaboration with cardiothoracic surgeons may aid in complex approaches traversing the thoracic cavity.

Resection of lumbar and sacral tumors necessitates close proximity to important vascular and gastrointestinal structures, and intraoperative injury to these structures can greatly exacerbate patient morbidity. Anterior approaches to lumbar vertebrae typically require mobilization of branches from the aorta and the inferior vena cava, risking significant hemorrhage [16]. Venous injury occurs more frequently than arterial injury and occurs in up to 15% of anterior exposures; the iliolumbar vein may overlie the L5 vertebral body and can be dissected to enhance exposure of the L4–5 disk space and to prevent traction-related tear of the left common iliac vein [17]. Collaboration with vascular, colorectal, and general surgeons is indicated to mitigate the risks of vascular or bowel injury and to aid in reconstruction [17,18].

### 3.3. Intraoperative Hemorrhage

Intraoperative hemorrhage is associated with significant morbidity and longer recovery times and is common in the surgical treatment of both metastatic and primary malignant spinal tumors [19]. More than 42% of patients who underwent en bloc resection of spinal tumors experienced massive blood loss greater than 5 L [20], and over one-third of patients undergoing intralesional resection of spinal metastases required perioperative blood transfusions [21,22]. Patients receiving intraoperative blood transfusions are further predisposed to postoperative infection and venous thromboembolism [19].

Careful preoperative evaluation is necessary to identify which patients are at greater risk of hemorrhage and likelier to require transfusions, as this evaluation can then inform perioperative management of fluid status and antibiotic course for high-risk patients. Mohme et al. analyzed 430 patients receiving oncologic spine surgery and devised a transfusion risk checklist which evaluates preoperative hemoglobin and ESCC score in addition to tumor histology [19].

Characterization of spinal tumors through tools like CT-guided biopsy and angiography can further stratify hemorrhage risk and inform surgical planning, as both primary and metastatic tumors can vary in vascularity [23,24,25]. Angiography additionally presents the opportunity for embolization of distal tumor vasculature to decrease the risk of volume loss associated with intraoperative hemorrhage [23], especially for the resection of hypervascular metastases such as those arising from multiple myeloma or from solid organs like the kidney and thyroid [19,26].

### 3.4. Cerebrospinal Fluid Leak

Cerebrospinal fluid (CSF) leaks are well-recognized complications of spine tumor surgery, leading to longer hospital stays, prolonged operation times, and higher healthcare costs [27]. CSF leaks may occur in 5 to 18% of cases and can result in further complications which impair patient recovery, including intracranial hypotension, wound dehiscence, durocutaneous fistulas, meningitis, tumor seeding along the tract of the leak, and revision surgery [28,29].

Avoiding unnecessary durotomy is critical. When durotomy is necessary for resection of intradural tumors, primary repair of the dura should be performed [28,30,31]. For large defects, auto- or allograft materials [32] or sealants [33] may be used to improve closure. Some authors utilize a small piece of surrounding muscle or fat to reinforce the closure [34]. An intraoperative Valsalva maneuver can be performed to identify persistent leaks. Following dural closure, fastidious wound closure is essential to prevent future CSF leak and re-operation [31]. The deep thoracodorsal fascia provides the most tensile strength and adequate closure of this layer is critical [7]. Collaboration with plastic surgeons to perform complex wound reconstruction may be considered for this purpose.

Postoperatively, conventional practice favors bedrest following durotomy for between 2 to 7 days [34], often with positioning restrictions that minimize CSF pooling at the site of the durotomy, such as flat for lumbar durotomy, and head of bed up for cervical surgery. These restrictions must be weighed against other post-operative risk optimization, as early ambulation may help prevent other postoperative complications such as venous thromboembolism [35,36,37,38], and being flat can increase risk of aspiration in extubation. Any drains that are left in durotomy cases must have output monitored for CSF. The duration of drainage and level of suction of the drain again presents a risk–benefit analysis: prolonged usage of drains to suction aids in avoiding postoperative seroma, hematoma, and optimizing superficial closure, but risks drawing CSF through the durotomy, disrupting the dural healing. Strategies to identify CSF in drain output include visual inspection and sampling for CSF markers such as beta-2 transferrin. Patients with asymptomatic durotomies may not require a prolonged bed rest or hospitalization and can be safely discharged home following surgery [39].

### 3.5. Wound Complications

Surgical site infections (SSI) can contribute to long-term antibiotic administration, additional surgeries, infection spread to the central nervous system, and ultimately perioperative morbidity and mortality. Patients with SSIs also have prolonged hospital length of stays and a higher rate of hospital readmission following discharge, leading to increased healthcare costs [40].

Patient risk factors for SSI following spine instrumentation include diabetes mellitus, hypertension, smoking history, and obesity; surgical risk factors for SSI include postoperative CSF leak, previous spinal surgery, and a higher number of instrumented levels [41,42]. Several factors predispose patients with spine tumors in particular to wound complications: this patient cohort often has a prior history of spine radiation and surgeries [41], and has a relative catabolic state [43], undermining their capacity for wound healing. Wound infection and breakdown following spinal neoplasm resection is common, occurring in one in ten patients [44]. This wound complication rate is higher than those observed in patients receiving spine surgery for non-oncological indications [45]. In a cohort of 159 patients undergoing metastatic spine tumor resection, researchers reported six reoperations related to wound dehiscence in addition to 16 reoperations for wound infections [43]. Preoperative stratification of patient-specific risk factors for surgical site infection and wound breakdown is critical to operative management of spine tumors. Optimization of risk factors for wound breakdown should involve smoking cessation, blood sugar control, blood pressure control, measuring acute phase reactants such as transferrin, prealbumin, albumin, and total lymphocyte count, and administering protein and vitamin supplementation to optimize nutrition and wound healing.

Special consideration should be given to patients with spine neoplasms that have undergone neo-adjuvant chemotherapy or radiation therapy. Literature has shown that these patients often have wound complications post-operatively [40,43,46]. These cytotoxic medications inhibit cell metabolism, cell division, and angiogenesis leading to impaired wound repair and may blunt the immune response to infection [47]. Expert consensus suggests that the interval between surgery and radiation therapy for spine tumors should be at least 2 weeks to avoid wound complications [43], although there is a paucity of literature on the subject [48]. Furthermore, the optimal treatment sequences of and interval between stereotactic radiosurgery and tumor resection is not known [49]. In these populations that are at a particularly high of post-operative SSI such as those with prior radiation or chemotherapy, plastic surgery closure may aid to attenuate the risks of infection following neoplasm resection [50].

### 3.6. Deep Venous Thrombosis and Pulmonary Embolism

Deep venous thrombosis (DVT) and pulmonary emboli (PE) are common, and often underdiagnosed, complications of patients with cancer [51]. Patients with malignancy have a 5 to 7 fold elevated risk for venous thromboembolism (VTE), owing to compression of venous valves as well as hemostatic alterations that occur in the setting of cancer [52,53]. Surgery itself further predisposes patients to VTE [54,55].

Despite this elevated risk, the ideal regimen and timing of chemoprophylaxis in spine surgery is controversial. Surgeons fear chemoprophylaxis will raise the risk for perioperative hemorrhage such as epidural hematoma and result in a poor neurological outcome. In one retrospective review of 6869 patients at a single center, the risks of spinal epidural hematoma among patients who receive chemoprophylaxis and those who do not are low and equivalent (0.2% vs. 0.18%, *p* = 0.622) [56].

The efficacy of chemoprophylaxis in preventing VTE in spine surgery is unclear—while most studies have found chemoprophylaxis reduces the incidence of VTE after spine surgery [57,58], some retrospective reviews suggest no difference in rate of VTE with or without chemoprophylaxis. Other studies even cite a higher rate among those who receive chemoprophylaxis than those who did not, though this likely represents selection bias, as the patients who received prophylaxis had more risk factors for VTE [56].

Mechanical VTE prophylaxis can be used as well: the largest systematic review on the topic found 70 studies of 16,164 high-risk patients and concluded that intermittent pneumatic compression devices reduced the rate of DVT from 16.7% to 7.3% and PE from 2.8% to 1.2% [59]. Similarly, early ambulation following surgery, compression stockings, and active and passive limb movement engender improved postoperative pain, shortened hospital length of stay, reduced opioid consumption, and ultimately improved functional outcome [60].

### 3.7. Pseudarthrosis

The long term goal of spinal fusion surgeries is an osseous union, and the failure of this process is known as pseudarthrosis. Pseudoarthrosis may be precipitated by hardware failure, wherein construct elements including rods, screws, and interbody cages fracture or migrate from their intended positions. Mechanical instability leading to progressive deformity and new or recurrent compression of neural elements may result and may ultimately require reoperation. Fusion in oncological spine surgery is complicated by tumor invasion and compromise of the native bone and the effects of radiation and chemotherapy on bony healing.

Fusion rates from resection of spinal column tumors vary from 36% to 100% [61]. Risk factors for pseudoarthrosis and hardware failure include three or more medical comorbidities, smoking, chest wall resection, and large construct lengths [62,63]. Additionally, special attention should be given to patients receiving pre-operative radiation or chemotherapy as these neo-adjuvant treatments have been shown to increase fusion and hardware failure rates [64]. Specifically, radiation therapy has been shown to cause dysvascular bone necrosis and fibrous replacement. This necrotic tissue may cause instability and provide a nidus for infection [65]. With regards to chemotherapy, certain agents have been shown to deplete bone marrow stromal progenitor cell populations decreasing osteogenesis [66]. The resulting osteopenia may engender poor pedicle screw fixation and cage subsidence [67,68], leading to fusion failure and revision surgeries [69].

A common mode of hardware failure is screw pullout, especially at the ends of a construct where stresses are greatest. When bone quality is poor, pullout strength of screws can be increased by injection of cement into vertebral bodies through a channel along the center of cannulated screws. This technique can ensure low rates of hardware failure in minimally invasive percutaneous approach when no tumor resection is required as well as in open surgeries involving destabilizing resections of tumors, such as corpectomy [70,71,72].

In larger open surgeries, stiffer constructs may be designed to better preserve spinal alignment. Whereas a typical fusion construct might have two rods posteriorly connecting screws on either side of the spine, additional rods may be added [71]. Retrospective studies comparing patients with non-oncological deformity undergoing long segment fusions with two-rod or multi-rod constructs showed increased rates of hardware failure in two rod constructs with greater rates of reoperation [73,74].

Finally, ensuring an appropriate cellular milieu to promote bone healing may be achieved through a variety of bone grafting strategies. The presence of viable osteoprogenitor cells is critical for integration of graft materials and successful bony remodeling. This may be achieved with autologous bone by harvest of material from a donor site outside of the spine or allograft mixed with bone marrow aspirate [75]. Whereas in non-oncological spine surgery bone from the spine that is removed during decompression can be utilized as autograft, this practice is not used during oncological surgery due to concern for local seeding with tumor cells. Structural grafts may also be used to restore the integrity of the anterior column. These include both vascularized strut grafts from, for example, an adjacent rib that remains connected to its vascular supply, or non-vascularized graft wherein morselized donor bone (either auto- or allograft) is packed into a synthetic cage [61,76].

As overall survival improves with improvements to local and systemic therapy for tumors metastatic to the spine, long-term complications of spinal fusion may occur in this population, including adjacent segment disease. Spinal fusion may change the forces applied to spinal segments not included in the fusion and hasten degenerative change at these levels. These changes may ultimately lead to secondary operations to address new symptomatic deformity. There exists minimal literature on this subject, but it will become an important consideration as patients and surgeons begin to confront the long-term consequences of spinal fusion.

## 4. Case Presentation

**Figure 1:**
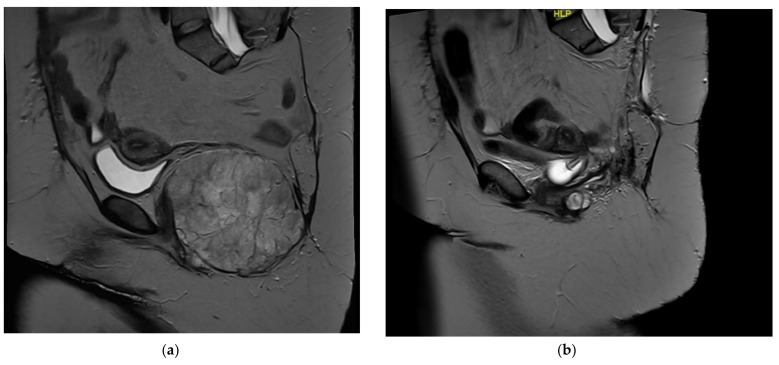
(**a**) Sagittal T2 MRI demonstrating a recurrent chordoma in the pelvic floor with evidence of prior sacrectomy. (**b**) Postsurgical sagittal T2 MRI demonstrating postsurgical changes after en bloc resection of chordoma requiring removal of the distal colon, rectum, and anus.

A 44-year-old female with a history of atrial fibrillation and recurrent sacral chordoma presents with two months of progressive right-sided foot drop, foot numbness, and radiating pain from the back down the posterior aspect of the right leg. Figure 1 displays preoperative imaging of the recurrent sacral chordoma. She previously underwent intralesional resection of a large sacral chordoma coupled with adjuvant stereotactic radiosurgery nine years prior, followed by high sacral amputation for a recurrent chordoma involving the right sciatic notch with bilateral preservation of the S1 and S2 nerve roots. The patient presented for palliative re-resection; ureteral stents were placed before a posterior approach was undertaken into the lumbosacral pelvic region for en bloc resection of multiple recurrent perirectal chordomas as well as resection of the patient’s anus, rectum, and sigmoid colon. A complex plastic closure of the buttock incision was performed using gluteal myocutaneous flaps. Perioperative antibiotics were given for 24 h. Chemical and mechanical DVT prophylaxis were initiated 24 h after the first stage. The patient’s postoperative course was complicated by brief urinary incontinence that spontaneously recovered and by wound dehiscence and infection requiring multiple operative debridements. A bilateral lower extremity ultrasound detected DVT in the right popliteal system, and the patient was started on therapeutic enoxaparin. The patient was discharged home with the wound vacuum, visiting nurse services, and a course of apixaban, and ultimately, she had a good functional outcome and resumed systematic therapy.

Although this patient’s wound complications were anticipated because of her significant radiation history, she also presented with histories of smoking and prior spinal surgery which increased her infection risk. In the event of surgical wound dehiscence and breakdown, patients should undergo incision debridement and receive empiric antibiotic therapy emergently. As observed in this case, patients with wound vacuums situated in the lumbopelvic region may be subject to prolonged bedrest and are at greater risk of VTE during their recovery.

## 5. Conclusions

Spinal tumors often do not respect anatomic boundaries, making complete resection complex and risking a range of medical and surgical complications. Providers should be aware of these complications to minimize risk and to identify them early in patient clinical courses. Table 1 documents rates of complication incidence following spinal tumor surgery reported in the literature. 

Understanding of anatomy and disease pathology can allow for complication risk mitigation in the perioperative period. Fastidious preparation for surgery including pre-operative medical optimization and imaging review, meticulous dissection informed by understanding of anatomic boundaries, and attentive medical care post-operatively can help avoid complications and improve patient outcomes. Table 2 summarizes the strategies discussed in this review for the prevention and management of complications in spine tumor surgery. The involvement of a multidisciplinary team is required to optimize outcomes for these patients.

## Figures and Tables

**Table 1:** Reported incidences for complications of spinal tumor surgery.

Complication	Reported Incidence
Intraoperative Hemorrhage	0.3–8.4% [77,78]
CSF Leak	6.6 ± 5.8% [28]
Wound Complications	7.1–9.6% [44,79]
Venous Thromboembolism	2.9% [80]
Pseudarthrosis	10.4–19.4% [61]

**Table 2:** Strategies for complication avoidance in spine tumor surgery.

Complication Type	Strategies for Prevention and Management
Approach-related Morbidity	**Preoperative**: − Occlusion testing to evaluate safety of potential vertebral artery sacrifice in cervical spine tumor surgery [14] **Intraoperative**: − Neuromonitoring and electrical nerve root stimulation [7] − Multidisciplinary surgical teams [7]
Intraoperative Hemorrhage	**Preoperative**: − Risk stratification using preoperative hemoglobin and tumor histology [19] − Angiography for assessment of tumor vascularity [23,24,25] − Embolization of distal tumor vasculture in hypervascular metastases [23] **Intraoperative**: − Collaboration with vascular surgery for vessel repair [22]
CSF Leak	**Intraoperative**: − Allograft/sealant placement following dural repair and closure [32,33] − Valsalva maneuver to identify persistent leaks [34] **Postoperative**: − 2–7 days of bedrest with positioning restrictions [34] − Meticulous inspection of drain output for CSF [34]
Wound Complications	**Preoperative**: − Risk factor optimization through smoking cessation and blood pressure control [41] **Intraoperative**: − Complex wound closure through collaboration with plastic surgery [46,50] **Postoperative:** − Recovery intervals greater than 2 weeks between surgery and adjunct radiation therapy [43]
Venous Thromboembolism	**Preoperative**: − No existing consensus on VTE chemoprophylaxis [56] **Postoperative**: − Chemical and mechanical VTE prophylaxis [59,60]
Pseudarthrosis	**Preoperative**: − Osteopenia assessment in patients with prior chemotherapy [66,67] **Intraoperative**: − Vertebral body cement injection through cannulated screws [70,71,72] − Multi-rod constructs to preserve spine alignment [73,74] − Vascularized strut grafts to bolster structural integrity [61,76]
